# Three-Year Follow-Up of Laser In Situ Keratomileusis Treatments for Myopia: Multi-Center Cohort Study in Korean Population

**DOI:** 10.3390/jpm11050419

**Published:** 2021-05-16

**Authors:** Jae-Yong Kim, Hun Lee, Choun-Ki Joo, Joon-Young Hyon, Tae-Im Kim, Jin-Hyoung Kim, Jin-Kuk Kim, Eun-Young Cho, Ji-Eun Choi, Na-Rae Lee, Hung-Won Tchah

**Affiliations:** 1 Department of Ophthalmology, Asan Medical Center, University of Ulsan College of Medicine, Seoul 05505, Korea; jykim2311@amc.seoul.kr (J.-Y.K.); yhun777@gmail.com (H.L.); 2 Department of Ophthalmology and Visual Science, Kangnam St. Mary’s Hospital, College of Medicine, The Catholic University of Korea, Seoul 06591, Korea; ckjoo8663@gmail.com; 3 Department of Ophthalmology, Seoul National University College of Medicine, Seoul National University Bundang Hospital, Seongnam 13620, Korea; jyhyon@snu.ac.kr; 4 Department of Ophthalmology, Institute of Vision Research, Corneal Dystrophy Research Institute, Yonsei University College of Medicine, Seoul 03722, Korea; taeimkim@gmail.com; 5 Department of Ophthalmology, Ilsan Paik Hospital, Inje University of Korea, Koyang 10380, Korea; jhk0924@hanmail.net; 6 B&VIIT Eye Center, Seoul 06615, Korea; bestjinkuk@gmail.com (J.-K.K.); doc-2000@hanmail.net (E.-Y.C.); 7 National Evidence-Based Healthcare Collaborating Agency, Seoul 04554, Korea; jechoi@neca.re.kr (J.-E.C.); cherishnr@neca.re.kr (N.-R.L.)

**Keywords:** laser in situ keratomileusis, efficacy, safety

## Abstract

This multi-center cohort study included 3401 myopic laser in situ keratomileusis (LASIK) procedures conducted in 1756 myopia patients between 2002 and 2005. Pre- and postoperative uncorrected visual acuity (UCVA), best corrected visual acuity (BCVA), and manifest refraction spherical equivalent (SE) were recorded. Factors predicting low postoperative efficacy (defined as a postoperative UCVA < 0.5) were identified using univariate and multivariate logistic regression analysis. Compared with 1 month postoperatively, logMAR UCVA at 3 months postoperatively was significantly decreased (*p* = 0.002) and that at 2 and 3 years was significantly increased (*p* < 0.001). LogMAR BCVA at 2 years postoperatively was significantly decreased compared with 1 month postoperatively (*p* = 0.008). Over the 3-year postoperative period, overall refractive predictability within ±1.00 D and ±0.50 D ranged from 69.0% to 86.2% and from 43.3% to 67.8%, respectively. This also decreased from 1 month to 6 months postoperatively (*p* < 0.005). Multivariate logistic regression analysis using generalized estimating equations, revealed that higher preoperative SE (odds ratio [OR], 2.58 and 7.23; *p* < 0.001) and lower preoperative BCVA (OR, 2.44; *p* = 0.003) were predictive of a low postoperative efficacy. In summary, myopic LASIK can be effective and safe with a high refractive predictability in a Korean population, but myopic regression occurs over time. Higher preoperative SE and lower preoperative BCVA are predictive of a low postoperative efficacy.

## 1. Introduction

Laser in situ keratomileusis (LASIK) was first introduced by Pallikaris et al. [1]. Since it was found to be highly effective and safe, resulting in a rapid vision recovery, and was associated with marginal patient discomfort, LASIK became the most common ophthalmic surgical procedure used worldwide [2,3,4,5,6]. Notably however, it involves a relatively long learning curve and is associated with the risk of flap-related complications such as free cap, incomplete flap, buttonholes, epithelial ingrowth, lost flaps, and deep lamellar keratitis [7,8,9,10,11,12].

In the initial LASIK case series in Korea reported in 1997, a nasal corneal flap of 130 or 160 µm in thickness was created using a mechanical microkeratome and ablation was performed with an MEL60 laser (Aesculap-Meditec, Germany) [13,14]. Previously, 100,000 LASIK surgeries were estimated to be performed in Korea every year. However, there has been a paucity of multi-center cohort studies of postoperative outcomes and complications of LASIK in Korean patients. Moreover, to our knowledge, the efficacy, safety, and predictability of LASIK has not been previously investigated in such a large series of Korean patients. We therefore conducted such analyses in a 2002–2005 Korean refractive surgery case series that had been followed up for three years. As we used the same cohort database, this study was an addendum of the prior study of Na et al. which showed that LASIK and surface ablation produced similar postoperative visual efficacy after corneal healing and that although the outcome predictability did not differ between the two procedures, myopic regression was observed more frequently in the surface ablation group [15]. The aim of the current study is to investigate the efficacy, safety, and predictability of LASIK using multi-center cohort data that included 3401 LASIK procedures conducted in 1756 myopia patients between 2002 and 2005.

## 2. Materials and Methods

The study samples were obtained from six ophthalmology centers, namely the Catholic University of Korea, the Yonsei University College of Medicine, the University of Ulsan College of Medicine (Asan Medical Center), the Seoul National University College of Medicine, the Inje University College of Medicine, and the B&VIIT Eye Center. The Institutional Review Board of each institute approved the study protocols (2009–0567).

The National Evidence-based Healthcare Collaborating Agency (NECA) reviewed the charts of 5109 eyes that underwent LASIK or surface ablation (including laser epithelial keratomileusis (LASEK), epi-LASIK, and photorefractive keratotectomy (PRK)) between 2002 and 2005. Inclusion criteria for the present study were as follows: (i) age 19–45 years, and (ii) presence of myopia with a manifest refraction spherical equivalent (MRSE) between −1.00 and −15.00 diopters (D). Patients were excluded from the analyses if they had previous ocular or intraocular surgery, evidence of acute or chronic corneal infection, corneal inflammation, glaucoma, amblyopia, retinal detachment, diabetic retinopathy, macular degeneration, or neuro-ophthalmic disease. A standardized case report form (CRF) was established for each patient, on which an experienced NECA investigator collected the following data from the medical chart: (1) preoperative data including age, sex, previous medical and surgical history, uncorrected visual acuity (UCVA), best corrected visual acuity (BCVA), manifest and cycloplegic refractions, slit-lamp examination, fundus examination, keratometry, intraocular pressure (IOP), pupil size, central corneal thickness (CCT), anterior chamber depth (ACD), white to white diameter measured with the corneal topography (ORBscan II; Bausch & Lomb, Inc., Rochester, NY), Schirmer test, and tear breakup time (TBUT); (2) surgical data including excimer laser and surgical type (LASIK, LASEK, PRK or epi-LASIK), hinge position, flap thickness and size, laser ablation time and depth, and postoperative eye-drops; (3) postoperative data including refractive error, UCVA, BCVA, IOP, CCT, keratometry, corneal topography, Schirmer test, and TBUT. The postoperative data were obtained at 1, 3, and 6 months and 1, 2, and 3 years. Of the original cohort of 5109 eyes (2638 patients), the 3401 eyes (1756 patients) that underwent LASIK were included in our current analyses.

The efficacy and safety indexes were calculated as follows: efficacy index = (postoperative UCVA/preoperative BCVA); safety index = (postoperative BCVA/preoperative BCVA). To ascertain the predictability of LASIK, the postoperative spherical equivalent (SE), topographical cylinder, and the frequencies of postoperative SE within ±0.50 D and ±1.00 D were investigated at all follow-up visits. The incidences of intraoperative and postoperative complications (including incomplete flap, buttonhole flap, epithelial ingrowth, retreatment, keratoectasia, dry eye syndrome, infectious keratitis, and corneal opacity) were determined. Keratoectasia was defined as an inferior topographic steepening of ≥5.0 D compared to the immediate postoperative appearance, the loss of two or more Snellen lines of UCVA, and a change in the manifest refraction of ≥2 D in either sphere or cylinder [16]. The presence or absence of corneal opacity was determined by slit-lamp microscopy performed at any follow-up visit.

In the present study, along with efficacy and safety indices, low postoperative efficacy was also defined as a postoperative UCVA that was poorer than 0.5 at any follow-up visit. Patient variables including age, preoperative IOP, SE, BCVA, CCT, and topography were analyzed for lower postoperative efficacy. Age and preoperative IOP, SE, and CCT were analyzed for postoperative corneal opacity. The cases were divided into three groups by age (≤29 years, 30–39 years, and ≥40 years) and were also stratified according to the level of preoperative myopia, i.e., low (preoperative SE < −6.00 D), moderate (preoperative SE between −6.00 D and −10.00 D), and high (preoperative SE > −10.00 D), and the preoperative topography, i.e., >43 D, 41–43 D, and <41 D. The subjects were further classified into two groups in accordance with preoperative intraocular pressure (<15 mmHg or ≥15 mmHg), preoperative logMAR BCVA (≤0 and >0), and preoperative CCT (≥500 µm and <500 µm).

### 2.1. LASIK Procedure Parameters

Table 1 showed the intraoperative parameters of LASIK procedures conducted in 3401 myopic eyes between 2002 and 2005. The B&L XP (Bausch & Lomb Surgical, Inc., Rochester, NY, USA), Moria M2 (Moria, Inc., Doylestown, PA, USA), Automated Corneal Shaper (ACS; Chiron Vision, Irvine, CA, USA) microkeratome, or the IntraLase femtosecond laser (Abbott Medical Optics, Inc., Santa Ana, CA, USA), was used to cut an anterior corneal flap of 8.5–9.75 mm in diameter which was superiorly-, nasally-, or temporally-hinged. A VISX 20/20 (Abbott Medical Optics, Inc., Santa Ana, CA, USA), Zioptix (Bausch & Lomb Surgical, Inc., Rochester, NY, USA), MEL80 (Carl Zeiss Meditec, Inc., Jena, Germany), or Allegretto (Alcon Laboratories, Inc., Fort Worth, TX, USA) excimer laser system was then used to ablate the stroma.

### 2.2. Statistical Analysis

The Kolmogorov-Smirnov test was used to confirm the normality of the data. Univariate and multivariate analyses using generalized estimating equations were subsequently performed to identify predictive factors for the low efficacy of the LASIK operation and postoperative corneal opacity. ANOVA was performed to compare the three myopia groups in terms of UCVA, efficacy index, BCVA, safety index, and topographical cylinder. The Chi-square test was used to compare the three myopia groups in terms of the percentage of eyes within ±0.50 D and ±1.00 D and the percentage of eyes with postoperative UCVA ≥ 20/40, 20/30, and 20/20. Correction for multiple comparisons between more than three groups was performed using Bonferroni adjustment. The statistical analyses were conducted using the SPSS^®^ program (version 25.0; IBM SPSS, Inc., Chicago, IL, USA) and the SAS program (version 9.1; SAS Institute, Inc., Cary, NC, USA). The level of significance was set at *p* < 0.05.

## 3. Results

A total of 3401 eyes of 1756 patients were analyzed in this study. The mean age of the patients at the time of LASIK surgery was 28.25 ± 6.24 years. There were 1297 females (73.9%) and 459 males. Table 2 lists the preoperative characteristics of the patients.

The overall logMAR UCVA was 1.02 ± 0.35 preoperatively. At 1 month, 3 months, 6-months, 1 year, 2 years, and 3 years postoperatively, this value was 0.02 ± 0.12, 0.02 ± 0.13, 0.03 ± 0.12, 0.03 ± 0.13, 0.05 ± 0.13, and 0.05 ± 0.14, respectively. Compared with the overall UCVA at 1 month postoperatively, that at 3 months was significantly decreased (*p* = 0.002), but that at 2 and 3 years postoperatively had significantly increased (*p* < 0.001).

**Figure 1:**
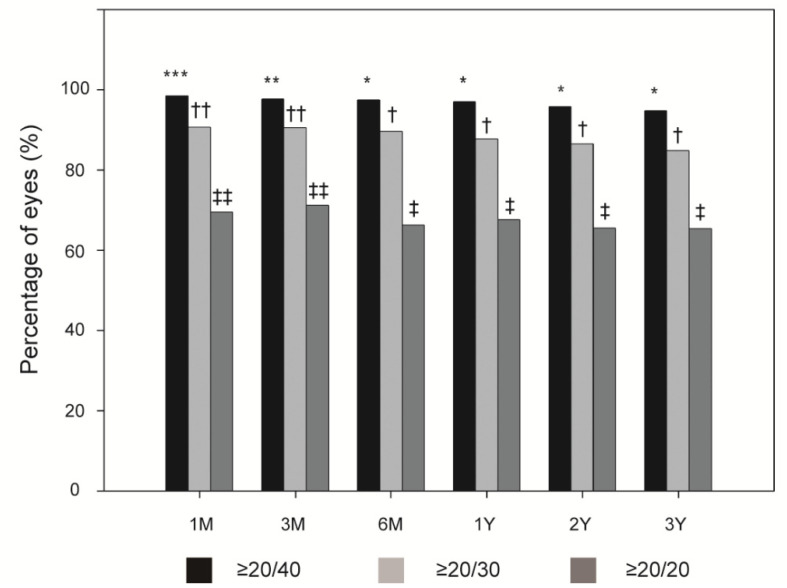
Overall incidence of postoperative uncorrected visual acuity ≥20/40, ≥20/30, and ≥20/20. ***, ** and *; †† and †; ‡‡ and ‡ indicate significant differences between these time points (all *p* < 0.005).

Figure 1 shows the overall incidences of postoperative UCVA ≥ 20/40, ≥20/30, and ≥20/20. At 1, 3, and 6 months, and 1, 2, and 3 years postoperatively, the overall efficacy index was 1.00 ± 0.29, 1.00 ± 0.36, 0.99 ± 0.25, and 1.00 ± 0.49, 0.98 ± 0.65, and 0.93 ± 0.25, respectively. A UCVA ≥ 20/40 had a significantly higher incidence at 1 month postoperatively than at 3 months postoperatively (all *p* < 0.005). Similarly, the overall postoperative UCVA ≥20/40 incidence was significantly higher at 1 and 3 months than at 6 months (all *p* < 0.005). This was also true for a UCVA ≥ 20/30 and 20/20 (all *p* < 0.005).

At 1, 3, and 6 months, and 1, 2, and 3 years postoperatively, overall logMAR BCVA was 0.02 ± 0.05, 0.02 ± 0.13, 0.03 ± 0.12, and 0.03 ± 0.13, 0.05 ± 0.13, and 0.05 ± 0.14, respectively. At the same time points, the overall safety index was 1.00 ± 0.22, 1.01 ± 0.26, 1.00 ± 0.11, and 1.02 ± 0.37, 1.05 ± 0.54, and 1.00 ± 0.11, respectively. The BCVA at 2 years postoperatively was significantly decreased compared with 1 month postoperatively (*p* = 0.008). Table 3 lists the type and incidence of LASIK-related complications. At 1 month postoperatively, 20 of 3401 eyes (0.6%) exhibited overcorrection, and 300 eyes (8.8%) exhibited undercorrection. Intraoperatively, an incomplete flap was found in four eyes (0.1%) and a buttonhole flap was detected in one eye. Postoperatively, epithelial ingrowth was observed in five eyes (0.1%). Re-treatment with laser ablation (enhancement) was performed in 23 eyes (0.7%). Furthermore, dry eye syndrome was found in 38 eyes (1.1%), infectious keratitis was observed in two eyes (0.1%), and corneal opacity was detected in 29 eyes (0.9%).

**Figure 2:**
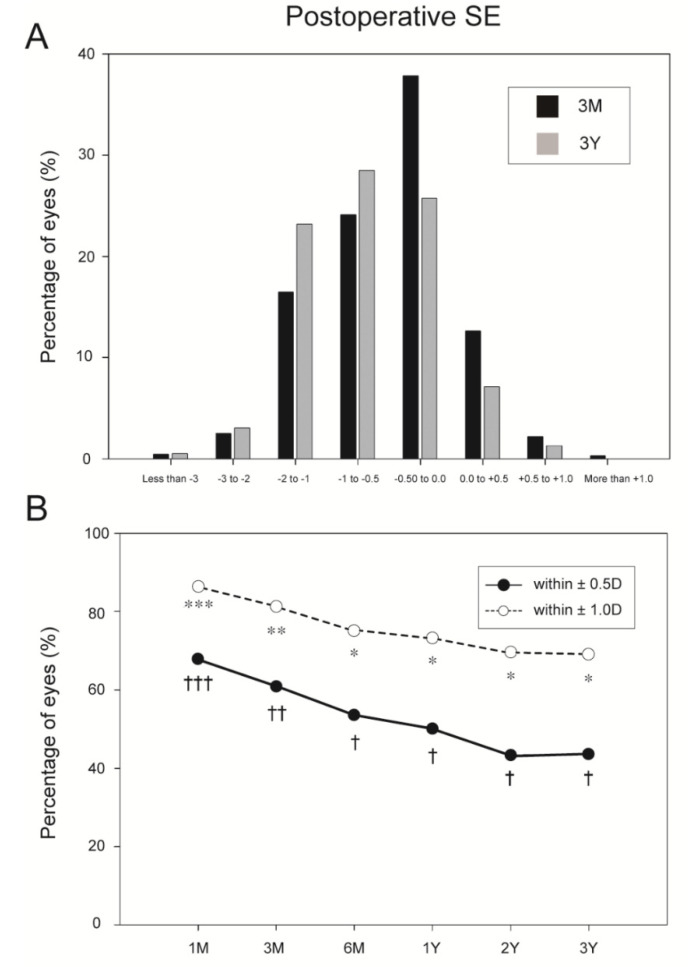
Distribution of postoperative spherical equivalents at 3 months and 3 years postoperatively (**A**) and percentage of eyes within ± 0.50 and ± 1.00 D emmetropia (in terms of spherical equivalent) at the indicated postoperative periods (**B**). ***, ** and *; †††, †† and † indicate significant differences between these time points (all *p* < 0.005). SE = spherical equivalent.

At 1, 3, and 6 months, and 1, 2, and 3 years postoperatively, SE was −0.31 ± 0.70, −0.46 ± 0.70, −0.55 ± 0.68, and −0.62 ± 0.66, −0.67 ± 0.64, and −0.72 ± 0.63, respectively. Compared with 1 month postoperatively, the SE values at 3 and 6 months, and 1, 2, and 3 years postoperatively were significantly decreased (all *p* < 0.001). Figure 2A shows the distribution of the postoperative SE at 3 months and 3 years postoperatively. The overall topographical cylinder was 1.40 ± 0.76 D preoperatively. At 1, 3, and 6 months, and 1, 2, and 3 years postoperatively, topographical cylinder was 1.06 ± 0.51 D, 1.06 ± 0.56 D, 1.05 ± 0.64 D, and 1.07 ± 0.56 D, 0.94 ± 0.47 D, and 1.02 ± 0.51 D, respectively. There were no significant differences between the topographical cylinder preoperatively and at any of the postoperative periods. During the 3-year follow-up period, the overall refractive predictability within ±1.00 D and ±0.50 D ranged from 69.0% to 86.2% and from 43.3% to 67.8%, respectively (Figure 2B). The overall refractive predictability within ±1.00 D and ±0.50 D decreased significantly from 1 to 6 months after the LASIK procedure (all *p* < 0.005). 

Univariate logistic regression analysis revealed that predictive factors for a low efficacy of LASIK included an age over 40 years (odds ratio [OR], 2.45; 95% confidence interval [CI], 1.21–4.94; *p* = 0.010; Table 4), a higher preoperative IOP (OR, 1.89; 95% CI, 1.09–3.27; *p* = 0.020), a higher preoperative SE (OR, 2.92 and 9.90; 95% CI, 1.76–4.86 and 4.10–23.91; all *p* < 0.001), and a lower preoperative logMAR BCVA (OR, 3.55; 95% CI, 2.06–6.14; *p* < 0.001). Furthermore, multivariate logistic regression analysis indicated that a low postoperative efficacy could be predicted by a higher preoperative SE (OR, 2.58 and 7.23; 95% CI, 1.53–4.33 and 2.74–19.11; all *p* < 0.001) and a lower preoperative logMAR BCVA (OR, 2.44; 95% CI, 1.37–4.37; *p* = 0.003; Table 5).

In terms of postoperative corneal opacity, univariate logistic regression analysis revealed that predictive factors for postoperative corneal opacity included a higher preoperative IOP (OR, 6.75; 95% CI, 1.59–28.57; *p* = 0.010) and a lower preoperative CCT (OR, 4.67; 95% CI, 1.73–12.63; *p* = 0.002; Table 6). In addition, postoperative corneal opacity was predicted by a higher preoperative IOP (OR, 8.49; 95% CI, 1.86–38.74; *p* = 0.006) and a lower preoperative CCT (OR, 7.36; 95% CI, 2.65–20.45; *p* < 0.001) according to multivariate logistic regression analysis (Table 7).

**Figure 3:**
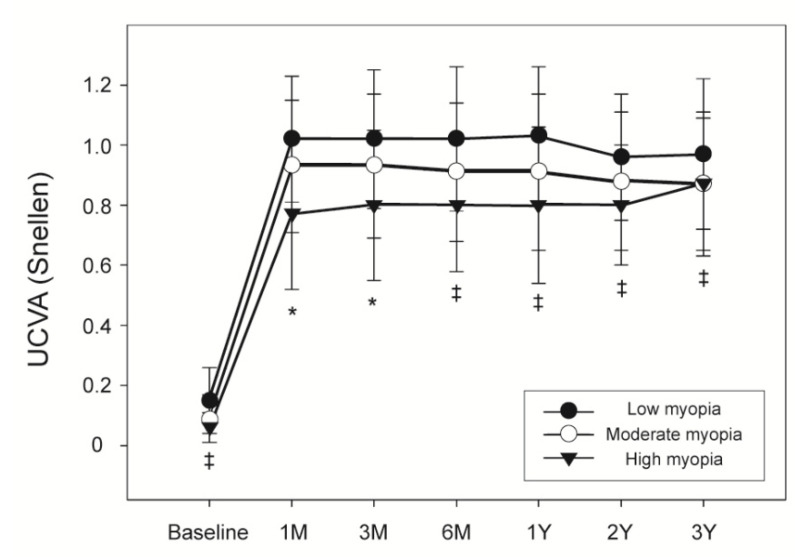
Comparison of pre- and postoperative uncorrected visual acuity according to the degree of myopia. *: significant difference among the three myopia groups (*p* < 0.050); ‡: significant difference between the low and moderate myopia groups, and between the low and high myopia groups (*p* < 0.050). UCVA = uncorrected visual acuity.

Since a lower preoperative SE was predictive of low postoperative efficacy, the eyes among our current study cohort were divided into three groups in accordance with the level of preoperative myopia. These three groups were then compared with regard to different variables. Figure 3 shows the preoperative and postoperative UCVA for these groups and how it changed between 1 month and 3 years after LASIK. The low myopia group had a significantly higher preoperative UCVA and also significantly higher UCVA values between 6 months and 3 years after LASIK than the other two groups. At 1 and 3 months postoperatively, there was a significant difference in UCVA among the three myopia groups. The severity of myopia at 1 and 3 months post-surgery correlated with a low postoperative UCVA.

**Figure 4:**
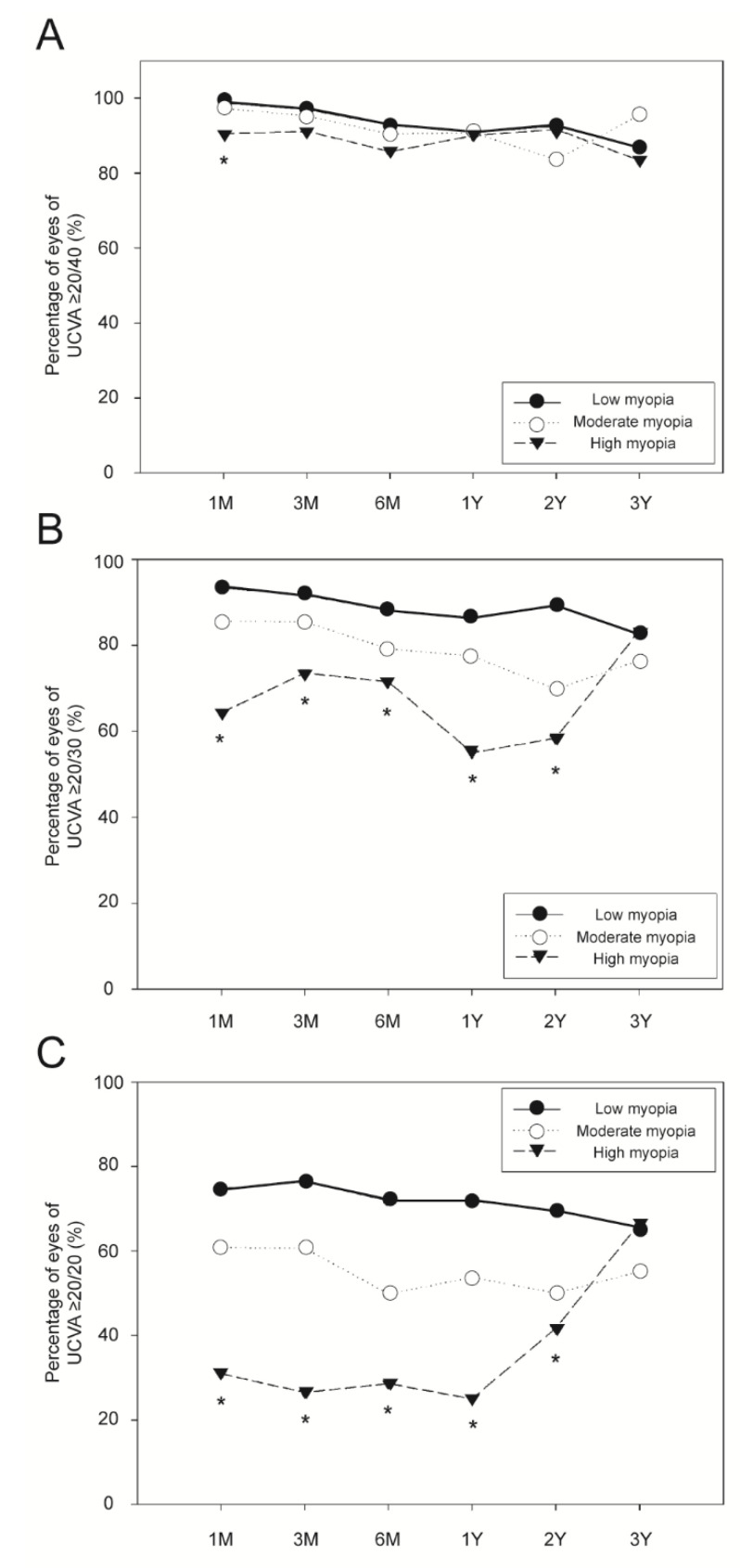
Incidence of uncorrected visual acuity of ≥20/40 (**A**), ≥20/30 (**B**), and ≥20/20 (**C**) according to the degree of myopia. *: significant difference among the three myopia groups (*p* < 0.010). UCVA = uncorrected visual acuity.

Figure 4 shows the incidences of a UCVA of ≥20/40, ≥20/30, and ≥20/20 in the three myopia groups during the postoperative period. In terms of the incidence of UCVA of ≥20/40, the three groups only differed significantly at 1 month postoperatively (*p* < 0.010; Figure 4A). The three groups differed significantly in terms of the incidences of UCVA of ≥20/30 and ≥20/20 at all follow-up periods except 3 years postoperatively (*p* < 0.010; Figure 4B,C).

**Figure 5:**
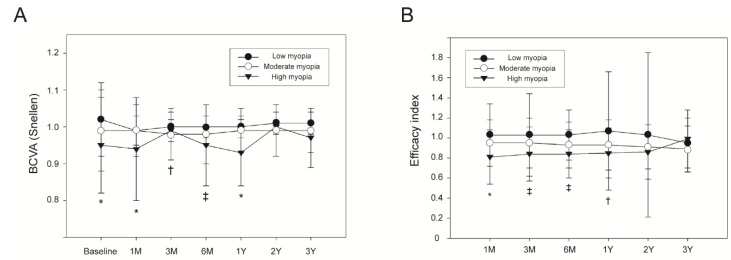
Comparison of best corrected visual acuity and efficacy index according to the degree of myopia. (**A**) BCVA. (**B**) efficacy index. *: significant difference among the three myopia groups (*p* < 0.050). †: significant difference between the low and moderate myopia groups (*p* < 0.050). ‡: significant difference between the low and moderate myopia groups, and between the low and high myopia groups (*p* < 0.050). BCVA = best corrected visual acuity.

Higher preoperative myopia was associated with a low preoperative BCVA and a low BVCA at 1 month and 1 year postoperatively (*p* < 0.050; Figure 5A). At 3 months postoperatively, BCVA was significantly higher in the low myopia group than in the moderate myopia group (*p* < 0.050). At 6 months postoperatively, BCVA was also significantly lower in the high myopia group than in the moderate or low myopia group (*p* < 0.050). Higher preoperative myopia was also significantly associated with a low postoperative efficacy at 1 month postoperatively (*p* < 0.050; Figure 5B). At 3 and 6 months postoperatively, the low myopia group had a significantly higher efficacy index than the moderate or high myopia groups (*p* < 0.050). At 1 year postoperatively, the low myopia group had a significantly higher efficacy index than the moderate myopia group (*p* < 0.050). The safety index of the three groups did not differ significantly at any time point.

**Figure 6:**
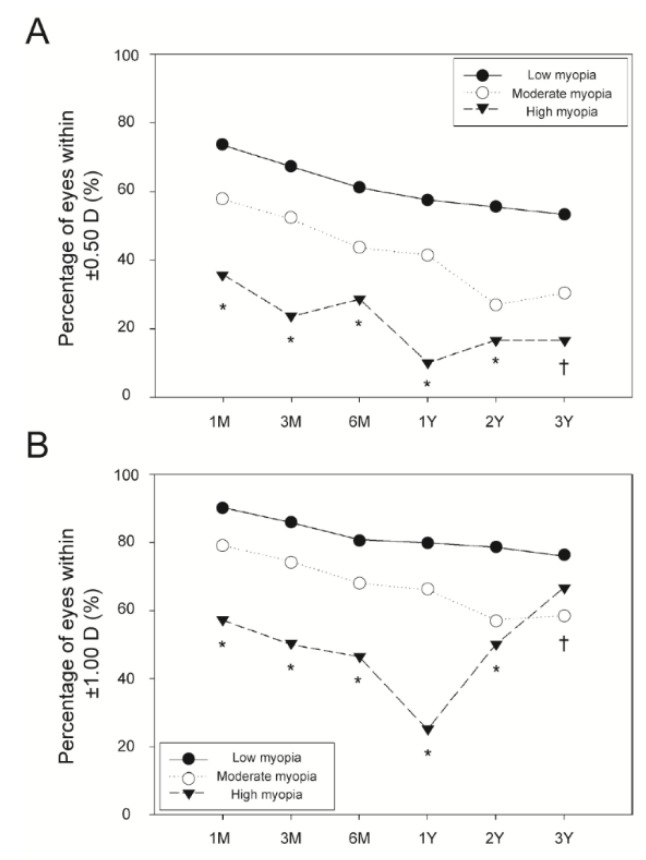
Refractive predictability assessed as a percentage of eyes within ±0.50 and ±1.00 diopters (D) of the postoperative spherical equivalent. These are separately shown as the percentage of eyes within ±0.50 D in the low, moderate, and high myopia groups (**A**) and as the percentage of eyes within ±1.00 D in these same three groups (**B**). *: significant difference among the three myopia groups (*p* < 0.010). †: significant difference between the low and moderate myopia groups (*p* < 0.050).

The three myopia groups differed significantly in terms of the refractive predictability within ±0.50 D and ±1.00 D (all *p* < 0.010; Figure 6A,B). However, they did not differ in terms of the postoperative SE. The low myopia group had a lower preoperative topographic cylinder than the other groups (*p* < 0.010). There were no differences between the three groups in terms of the topographic cylinder after surgery.

## 4. Discussion

In the present study, we evaluated the three-year follow-up of LASIK treatments for myopia in the Korean population and demonstrated that myopic LASIK can be effective and safe with a high refractive predictability, albeit myopic regression occurs over time.

Asians are reported to have a higher incidence of high myopia than Caucasians [17,18,19,20]. Several previous studies have shown that LASIK can be an effective keratorefractive surgical procedure for Asian eyes as it is associated with fast visual improvement and minimal pain and discomfort. A recent study using single-center cohort data that included 53,731 LASIK procedures conducted in 27,312 myopia patients between 1998 and 2015, showed that myopic LASIK performed in Asian (Chinese) eyes is safe and effective, with high refractive predictability [21]. Li et al. also found that thin-flap LASIK using a femtosecond laser or a mechanical microkeratome could safely and effectively correct high myopia in Chinese eyes [22]. However, Asano-Kato et al. suggested that the higher incidence of insufficient fixation of the microkeratome in LASIK might relate to the narrow palpebral fissures in Asian eyes [23]. Furthermore, Albietz et al. have reported that Asian eyes had a significantly higher rate of chronic dry eye after LASIK than Caucasian eyes, probably because the attempted refractive correction was higher in Asian eyes and there were also racial differences in terms of eyelid and orbital anatomy, tear file parameters, and blinking dynamics [24]. It is noteworthy that our present study is the first to analyze a multi-center cohort of Korean eyes that had undergone myopic LASIK and were followed up for a long period of 3 years. Another advantage of this study was that all of the selected subjects were Koreans, which could control for possible ethnic variations in the findings.

Previous large studies of LASIK for myopia have reported that 97–99% of the treated eyes achieved a postoperative UCVA ≥20/40, and 62–73% achieved one of ≥20/20 [21,25,26,27,28]. According to a recent study by the Singapore National Eye Center which involved an eighteen-year prospective audit of LASIK outcomes for myopia in 53,731 eyes, the overall efficacy index was 0.91, with >99% of eyes achieving UCVA of ≥20/40 and >70% achieving 20/20 [21]. Although the current study differed from those previous reports in terms of the follow-up period and preoperative refraction levels, our efficacy outcomes were similar, i.e., 3 months after LASIK, 96.3% of our cases achieved a UCVA of ≥20/40 and 69.9% achieved a UCVA of ≥20/20.

Previous Food and Drug Administration (FDA) clinical trials in the United States have shown that when eyes with low myopia (defined by a preoperative SE of less than −6.0 D) are treated with conventional LASIK, 67–86% of the cases achieve a UCVA of ≥20/20 and 93–100% achieve one of ≥20/40 [25,29]. Similarly, in the current study, 76.4% of the low myopia eyes achieved a UCVA of ≥20/20, and 97.0% achieved a UCVA of ≥20/40 by 3 months postoperatively. With regard to eyes with moderate myopia (defined as a preoperative SE of −6.0 to −12.0 D), published trials have reported that 26–77% of these eyes achieve a UCVA of ≥20/20 [30,31,32]. Our present classification of moderate myopia differed somewhat from this (we defined this as a preoperative SE of –6.0 to –10.0 D), but we obtained similar results at 3 months postoperatively; 60.7% of the moderate myopic eyes in our cohort achieved a UCVA of ≥20/20. In addition, 95.3% of these eyes achieved a UCVA of ≥20/40 at 3 months after LASIK. With regard to the high myopia group in the current study (which we defined as a preoperative SE of −10.0 D or higher), the LASIK efficacy was poorer than for the other two groups, as only 26.5% of these eyes achieved a UCVA of ≥20/20, although 91.2% achieved a UCVA of ≥20/40. In previous reports on cases with more severe myopia, higher ablation was followed by more wound healing and myopic regression, and surgeons seemed to perform undercorrection when faced with limits imposed by corneal thickness [28,32,33].

The overall efficacy indices in the current study during the 3-year follow-up exceeded 0.95, which is higher than the efficacy indices of 0.86–0.91 that were reported by previous studies [5,21,28]. These good outcomes may reflect the fact that the laser systems and ablation nomograms had improved and that most of the surgeons were already experts. With regard to the overall refractive predictability, FDA trials have reported that the predictabilities within ±1.00 D and ±0.50 at 3 months postoperatively were 90% and 72%, respectively [25,29]. The Singapore National Eye Center trial reported more than 94.0% of eyes achieved within ±1.0 D of target refraction, and at least 70% achieved within ±0.50 D of target at 3 months postoperatively [21]. Moreover, O’Doherty et al. reported that the predictabilities within ±1.00 D and ±0.50 D at 2 months postoperatively were 81.0% and 67.0%, respectively [34]. This latter study also reported that there was a postoperative regression toward myopia, as there was a mean change in refraction of −0.50 D over 5 years for all eyes, but the severely myopic eyes had a stronger regression with a mean change of −1.06 D [34]. In a series of 729 eyes, Kato et al. reported predictabilities within ±1.00 D and ±0.50 D at 3 months postoperatively of 93.1% and 82.6%, respectively [35]. Our current results are similar to what has been reported in the aforementioned studies. We also observed that the overall refractive predictabilities within ±1.00 D and ±0.50 D decreased significantly over time.

Few studies to date have examined the long-term stability of LASIK. Ikeda et al. reported that LASIK offered good safety outcomes during their 12-year observation period, but the efficacy and predictability gradually decreased with time because of myopic regression [32]. Kato et al. reported that postoperative refraction regressed minimally but significantly 1 year after LASIK [35]. Alio et al. showed that the mean post-LASIK refraction decreased slightly over 10 years; in eyes with up to −10 D myopia, the mean myopic regression was −0.12 D per year, while in eyes with myopia exceeding −10 D, this regression was −0.25 D per year [5,6]. In the current study, the post-LASIK refraction also regressed significantly over time.

It is noteworthy that our present multivariate logistic regression analysis revealed that high myopia was a risk factor for low postoperative efficacy. Megallanes et al. also found that extremely myopic eyes (<−15.0 D) exhibited a greater myopic shift and keratometric increase at 1 year after LASIK than highly myopic eyes (≥−15.0 D) [36]. When Rosman et al. followed up 141 eyes with myopia exceeding −10 D for at least 10 years after undergoing LASIK, they found that only 45.5% had a UCVA of 20/40 or better, and that the safety index was 0.87 at 10 years postoperatively [37]. In contrast, Bailey et al. reported that the postoperative UCVA and refraction did not differ among high myopia, low–moderate myopia, spherical myopia, and myopic astigmatism subgroups, and resembled the values of the entire cohort [25].

Despite the many studies that support the efficacy of LASIK, concerns remain about its possible long-term complications, including iatrogenic keratoectasia with progressive myopia [38,39,40]. After LASIK surgery, the cornea is permanently and structurally altered, not only by the laser ablation (depending on the attempted correction), but also by the creation of the flap itself [41]. Thus, the possibility of chronic stromal remodeling, unstable corneal biomechanics, and late regression remains [42]. One study has reported that refractive and topographic stability are achieved by 6 months after LASIK [43]. In the current study, no cases of keratoectasia were reported. This may reflect the meticulous preoperative patient selection that was performed at our six participating centers to reduce the risk of this complication. Moreover, the 3-year follow-up period was sufficient to observe the incidence of post-LASIK keratectasia, as it is reported to show immediately or many months after LASIK but generally within 2 years of surgery [44,45].

It was of interest that a higher preoperative IOP and lower preoperative CCT were found to be predictive of postoperative corneal opacity. There were previous articles that reported stromal haze after LASIK, which is associated with wound-healing reactions from significant keratocyte activation in the central flap stroma and interface [46,47]. Depending on its onset, severity, and duration, there are several differential diagnoses of corneal opacity, including diffuse lamellar keratopathy (DLK) [48,49,50,51], central toxic keratopathy (CTK) [52,53,54], and subepithelial opacity due to a thin LASIK flap [55]. However, the causes of the corneal opacity in the 29 cases in our study could not be determined due to a lack of information in the medical records. However, a previous report on another LASIK case series from Korea observed a DLK incidence of 0.2% (1 eye) [56]. Moreover, when Sander et al. performed a retrospective multi-center chart review of LASIK for myopia of between –4.00 and –7.88 D, with a 6-month follow-up, they found a DLK incidence of 4.8% (81 eyes) [57]. Corneal thinning after LASIK has also been associated with a severe form of DLK [58] that has been suspected in other reports to correspond to a different disease entity, namely CTK [52,53,59].

Our present study had several limitations. First, it may have had various issues associated with its retrospective design, including a substantial amount of missing data from the follow-up examinations, inter-observer bias, and selection bias. Second, because it was a multicenter study, there was no control over the number or uniformity of surgeons or technicians performing the preoperative examinations. For example, the excimer laser system used for laser ablation, specific skills, preferred operative methodology or nomograms might have differed among surgeons. Third, the corneal opacity cases could not be diagnosed in detail because of a lack of information in the medical records.

## 5. Conclusions

LASIK is an effective and safe intervention for a Korean population with myopia. Furthermore, our present findings can also serve as preliminary data in the development of guidelines for myopic LASIK surgery in the Asian population.

## Figures and Tables

**Table 1:** Intraoperative parameters (n = 3401).

Parameter	Mean ± SD	Range
Flap size (mm)	8.70 ± 0.53	8.50 to 9.75
Flap thickness (µm)	125.70 ± 23.43	59 to 200
Actual correction (D)	−4.98 ± 1.95	−11.75 to −0.13
Optical zone diameter (mm)	6.00 ± 6.39	4.7 to 7.5
Abrasion depth (µm)	71.45 ± 23.05	6 to 154
Position of flap		
Superior, n (%)	2654 (78.0)	
Nasal, n (%)	274 (8.1)	
Temporal, n (%)	3 (0.1)	
Unchecked, n (%)	470 (13.8)	
Keratome		
Moria M2, n (%)	1015 (29.8)	
IntraLase, n (%)	990 (29.1)	
Hansatome, n (%)	760 (22.3)	
Automated Corneal Shaper, n (%)	109 (3.2)	
Others, n (%)	527 (15.6)	
Laser platform		
VISX, n (%)	1077 (31.7)	
Zyoptix, n (%)	823 (24.2)	
MEL80, n (%)	465 (13.7)	
Allegretto, n (%)	149 (4.3)	
Unchecked, n (%)	887 (26.1)	
Wavefront-guided LASIK, n (%)	989 (29.1)	

D, diopters; LASIK, Laser in situ keratomileusis.

**Table 2:** Preoperative characteristics of eyes (n = 3401).

Parameter	Mean ± SD	Range
LogMAR UCVA	1.02 ± 0.35	0.1 to 2.3
LogMAR BCVA	0.00 ± 0.05	−0.3 to 1
IOP (mmHg)	15.38 ± 2.95	6 to 30
Spherical equivalents (D)	−4.96 ± 2.02	−12.75 to 0
Corneal thickness (µm)	546.62 ± 33.29	386 to 664
Mean keratometry (D)	43.42 ± 1.36	39.1 to 49.5
ACD (mm)	3.17 ± 0.78	2.4 to 4.7
White to white diameter (mm)	11.48 ± 0.37	10.1 to 13.4
Pupil size (mm)		
Scotopic	6.51 ± 0.76	3.7 to 8.7
Photopic	4.10 ± 0.76	0.7 to 7.9
Dry eye syndrome		
Yes, n (%)	1753 (77.5)	
No, n (%)	509 (22.5)	
Retinal abnormality		
Normal, n (%)	2993 (88.0)	
Peripheral tear, n (%)	5 (0.1)	
Peripheral degeneration, n (%)	132 (3.9)	
Others, n (%)	73 (2.1)	

UCVA, uncorrected visual acuity; BCVA, best corrected visual acuity; IOP, intraocular pressure; D, diopters; ACD, anterior chamber depth.

**Table 3:** Incidence of laser in situ keratomileusis complications (n = 3401).

	n (%)
Intraoperative complications	
Incomplete flap	4 (0.1)
Buttonhole flap	1 (0.0)
Postoperative	
Epithelial ingrowth	5 (0.1)
Re-treatment	23 (0.7)
Corneal ectasia	0 (0.0)
Miscellaneous	
Dry eye syndrome	38 (1.1)
Infectious keratitis	2 (0.1)
Postoperative corneal opacity	29 (0.9)

**Table 4:** Univariate logistic regression analysis to assess the predictor variables of low postoperative efficacy.

Parameter	OR	95% Confidence Interval	*p*
Age, y			
≤29	1.00		
30–39	0.82	0.46 to 1.46	0.490
≥40	2.45	1.21 to 4.94	0.010
Preoperative IOP (mmHg)			
<15	1.00		
≥15	1.89	1.09 to 3.27	0.020
Preoperative SE (D)			
<−6.0	1.00		
between −10.0 and −6.0	2.92	1.76 to 4.86	<.001
>−10.0	9.90	4.10 to 23.91	<0.001
Preoperative logMAR BCVA			
≤0.0	1.00		
>0.0	3.55	2.06 to 6.14	<0.001
Preoperative CCT (µm)			
≥500	1.00		
<500	4.67	1.73 to 12.63	0.002
Preoperative mean keratometry (D)			
>43	1.00		
Between 41 and 43	0.99	0.61 to 1.63	0.980
<41	0.60	0.14 to 2.49	0.480

OR, odds ratio; IOP, intraocular pressure; SE, spherical equivalent; D, diopters; BCVA, best corrected visual acuity; CCT, central corneal thickness.

**Table 5:** Multivariate logistic regression analysis to assess the predictor variables of low postoperative efficacy.

Parameter	OR	95% Confidence Interval	*p*
Age, y			
≤29	1.00		
30–39	0.80	0.44 to 1.45	0.460
≥40	2.01	0.97 to 4.16	0.060
Preoperative IOP (mmHg)			
<15	1.00		
≥15	1.70	0.94 to 3.06	0.080
Preoperative SE (D)			
<−6.0	1.00		
between -10.0 and−6.0	2.58	1.53 to 4.33	<0.001
>−10.0	7.23	2.74 to 19.11	<0.001
Preoperative logMAR BCVA			
≤0.0	1.00		
>0.0	2.44	1.37 to 4.37	0.003
Preoperative CCT (µm)			
≥500	1.00		
<500	1.85	0.60 to 5.68	0.280
Preoperative mean keratometry (D)			
>43	1.00		
Between 41 and 43	1.10	0.66 to 1.83	0.720
<41	0.68	0.17 to 2.79	0.590

OR, odds ratio; IOP, intraocular pressure; SE, spherical equivalent; D, diopters; BCVA, best corrected visual acuity; CCT, central corneal thickness.

**Table 6:** Univariate logistic regression analysis to assess the predictor variables of postoperative corneal opacity.

Parameter	OR	95% Confidence Interval	*p*
Age, y			
≤29	1.00		
30–39	1.64	0.73 to 3.69	0.230
≥40	0.55	0.07 to 4.07	0.560
Preoperative IOP (mmHg)			
<15	1.00		
≥15	6.75	1.59 to 28.57	0.010
Preoperative SE (D)			
<−6.0	1.00		
between −10.0 and −6.0	1.86	0.85 to 4.09	0.120
>−10.0	3.30	0.44 to 24.89	0.250
Preoperative CCT (µm)			
≥500	1.00		
<500	4.67	1.73 to 12.63	0.002

OR, odds ratio; IOP, intraocular pressure; SE, spherical equivalent; D, diopters; CCT, central corneal thickness.

**Table 7:** Multivariate logistic regression analysis to assess the predictor variables of postoperative corneal opacity.

Parameter	OR	95% Confidence Interval	*p*
Age, y			
≤29	1.00		
30–39	1.65	0.73 to 3.69	0.230
≥40	0.75	0.10 to 5.66	0.780
Preoperative IOP (mmHg)			
<15	1.00		
≥15	8.49	1.86 to 38.74	0.006
Preoperative SE (D)			
<−6.0	1.00		
between −10.0 and −6.0	2.08	0.95 to 4.56	0.070
>−10.0	4.11	0.51 to 32.99	0.180
Preoperative CCT (µm)			
≥500	1.00		
<500	7.36	2.65 to 20.45	<0.001

OR, odds ratio; IOP, intraocular pressure; SE, spherical equivalent; D, diopters; CCT, central corneal thickness.

## Data Availability

Data are available upon request from the authors.
